# Supplementary figures and images for: Chemical disruption of ABA signaling overcomes high-temperature inhibition of seed germination and enhances seed priming responses
Source: PLoS One. 2024 Dec 11;19(12):e0315290. doi: 10.1371/journal.pone.0315290 (PMC11634006; doi:10.1371/journal.pone.0315290)

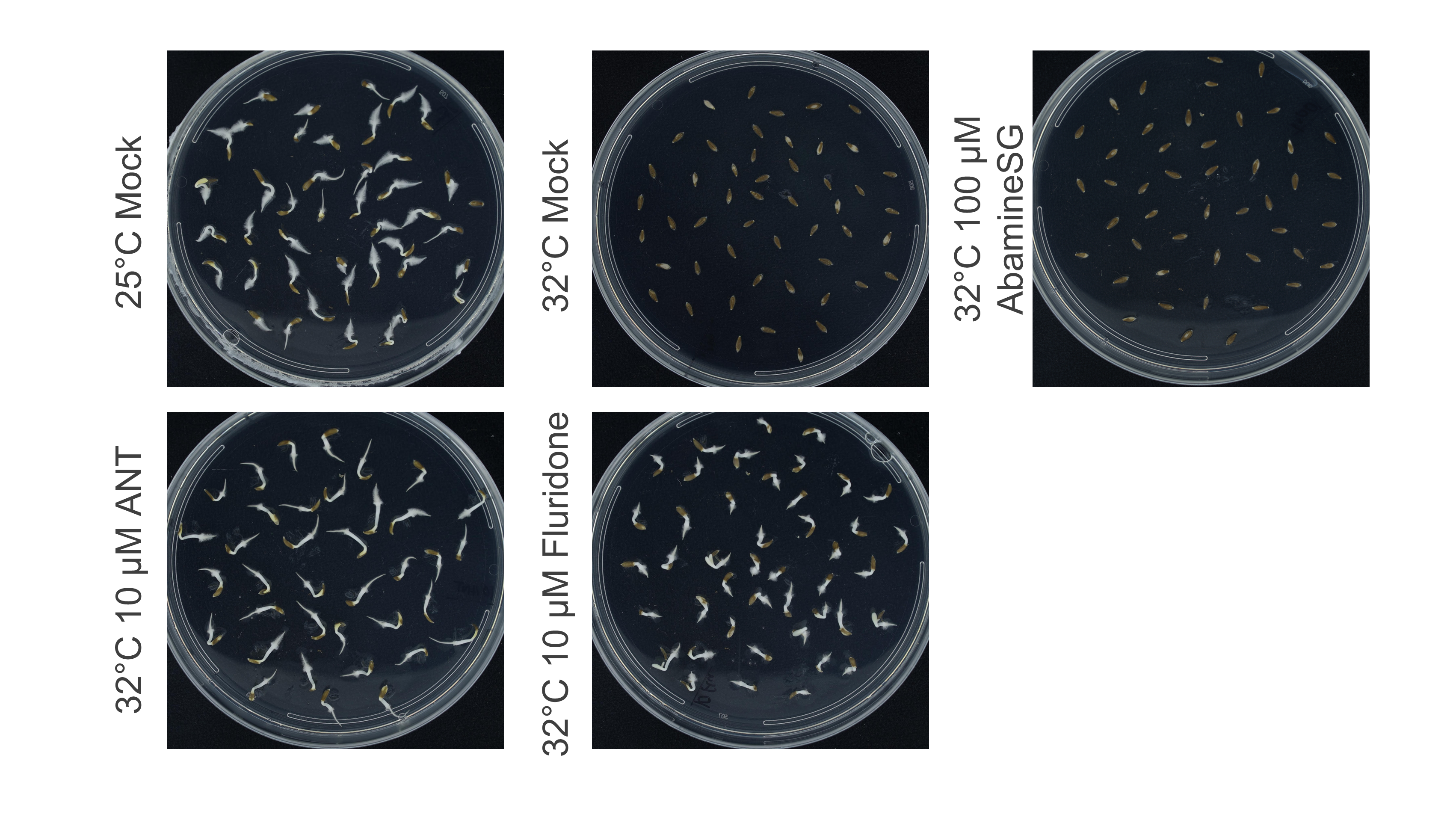

Supplement: S1 Fig — Germination was quantified for seeds imbibed on plates containing DMSO (mock-treated), 10 μM ANT, 10 μM fluridone or 100 μM AbamineSG and grown for 48h. Representative images are shown. (TIF) [file pone.0315290.s001.TIF]

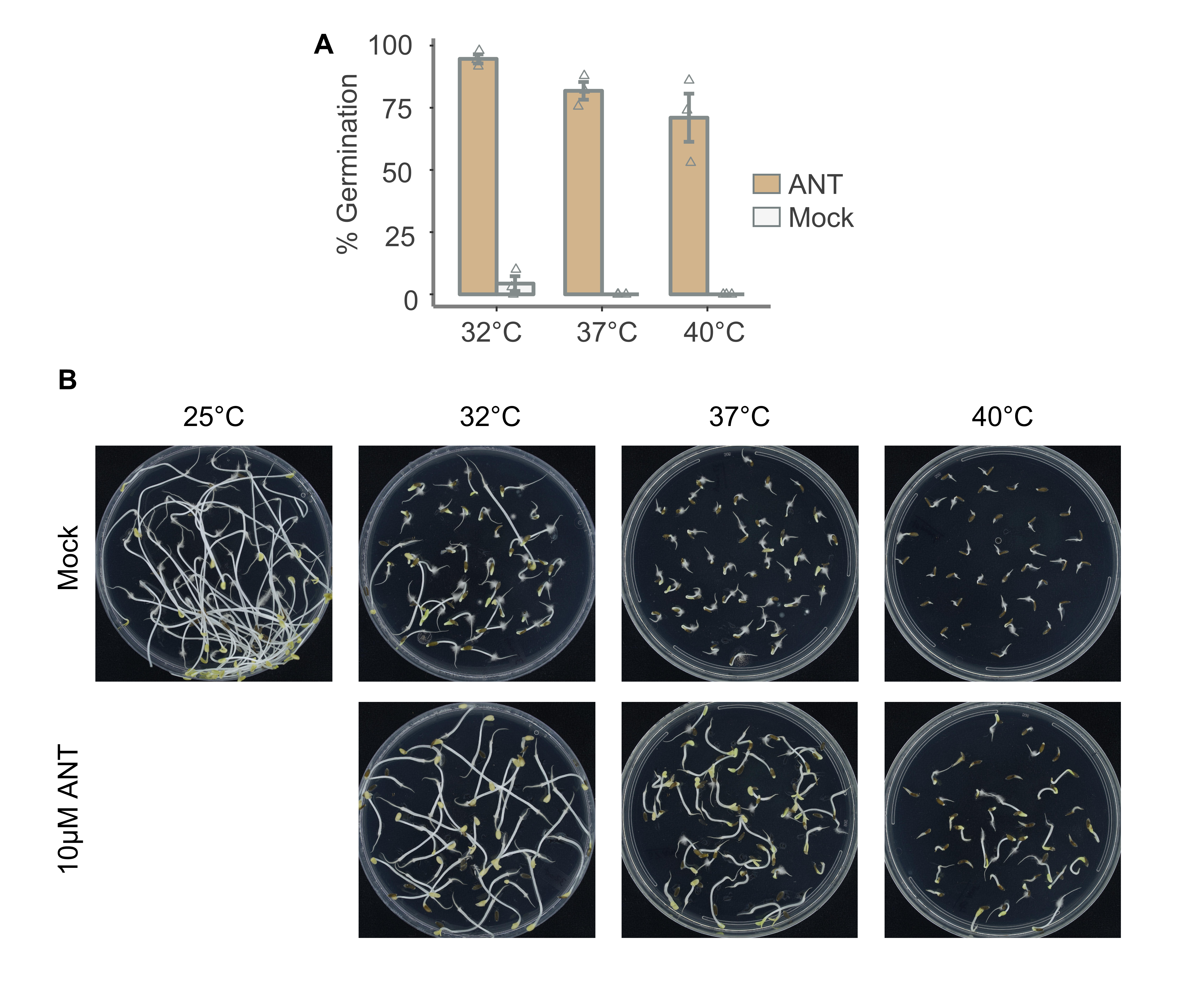

Supplement: S2 Fig — (A) Seeds treated with 10 μM ANT begin to germinate at temperatures of up to 40°C after 12048 hours of imbibition. (B) After 120 hours of heat treatment and 72 hours of recovery in dark at 25°C, seeds germinate. (TIF) [file pone.0315290.s002.TIF]

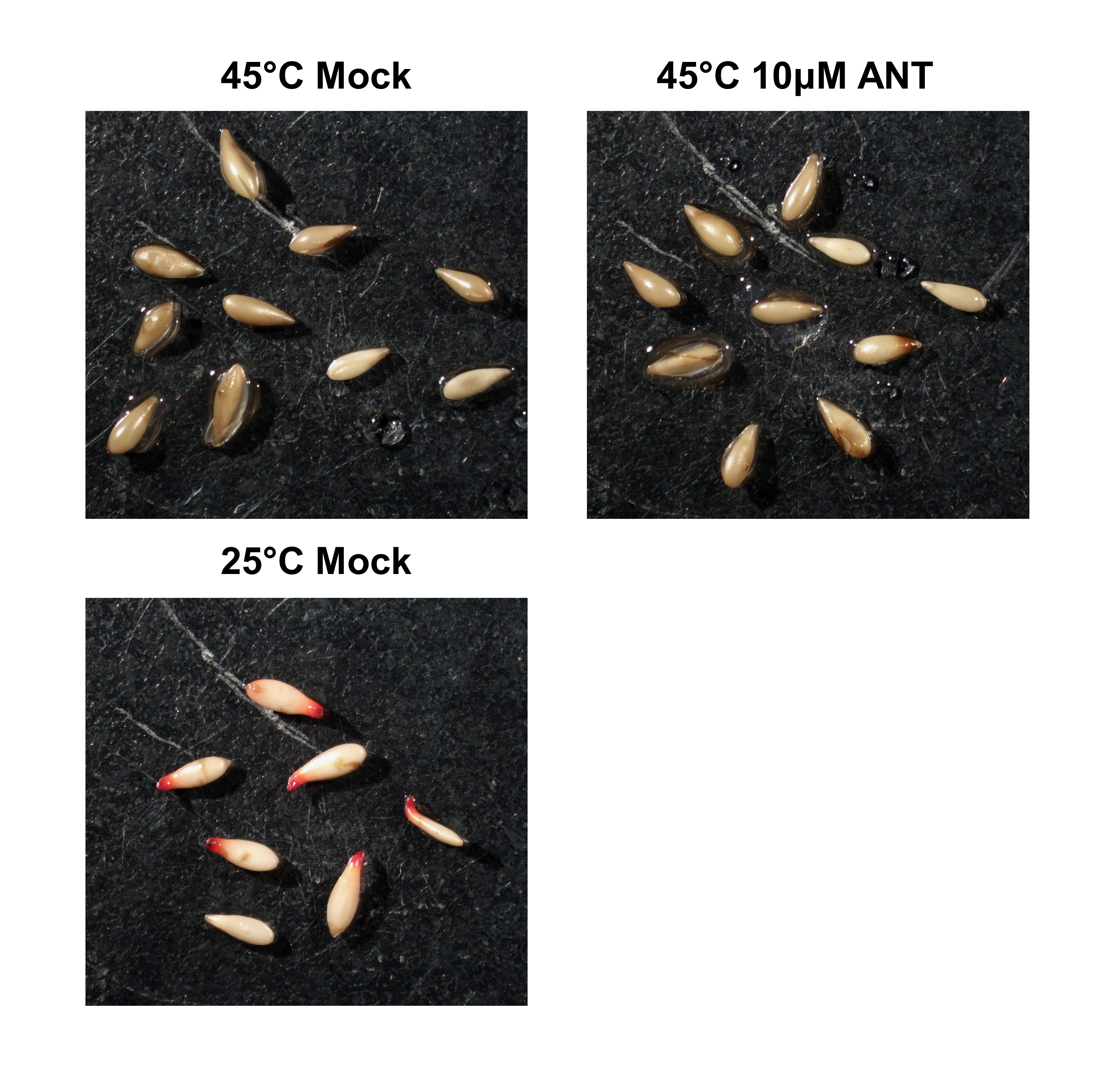

Supplement: S3 Fig — Seeds were imbibed in dark in 10 μM ANT or mock treatment at 45°C for 120 hours before tetrazolium staining. These seeds were compared to mock-treated seeds grown in the dark for 24 hours before staining. (TIF) [file pone.0315290.s003.TIF]

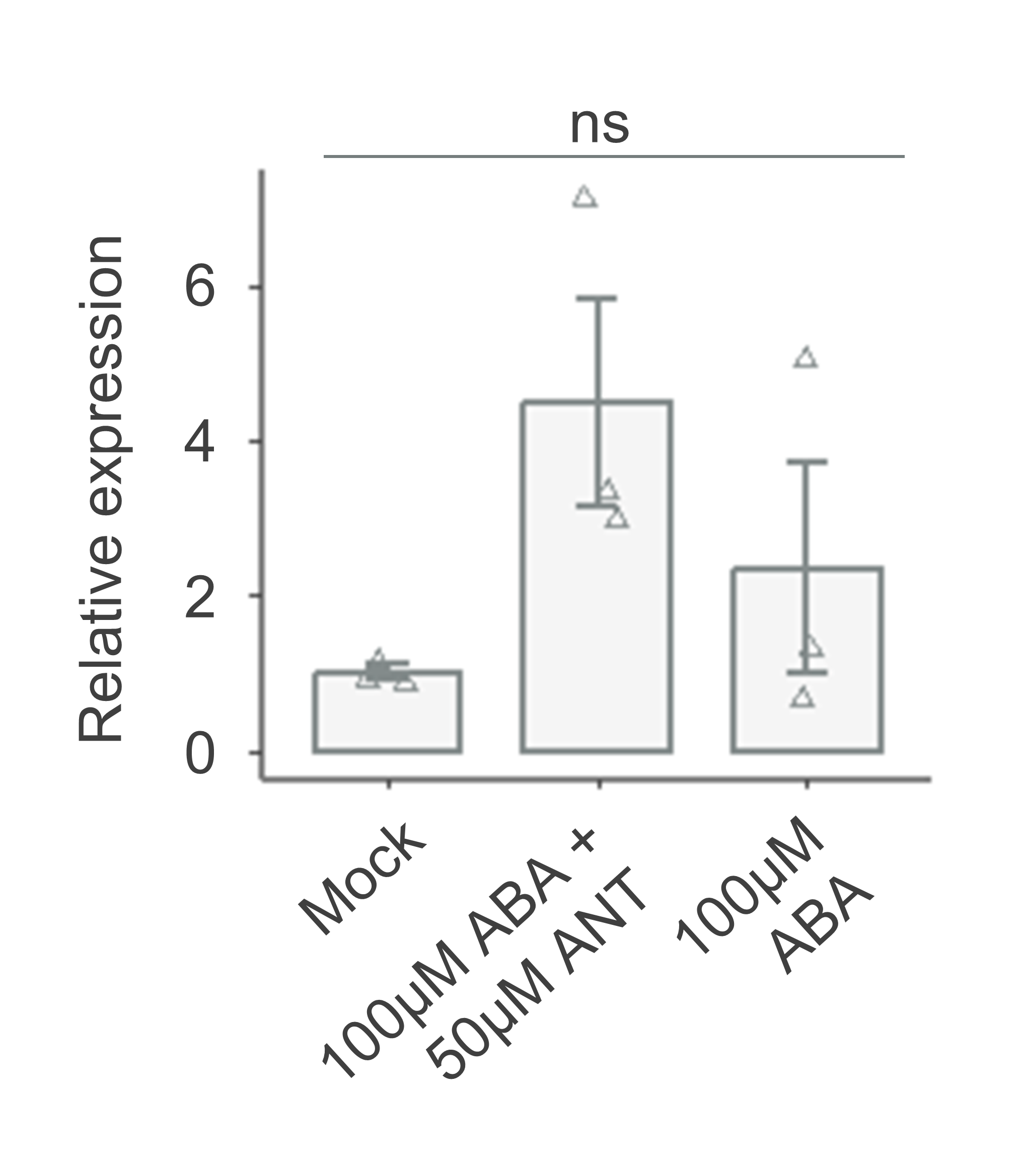

Supplement: S4 Fig — ABA and ABA + ANT treatments do not alter LsNCED4 expression (normalized to UBC21) measured by qRT-PCR of lettuce leaves in (DMSO) or 100 μM ABA or 100 μM ABA and 50 μM ANT in water (pairwise t test, p>0.05). (TIF) [file pone.0315290.s004.TIF]
