# Supplementary material for: Chemical disruption of ABA signaling overcomes high-temperature inhibition of seed germination and enhances seed priming responses
Source: PLoS One. 2024 Dec 11;19(12):e0315290. doi: 10.1371/journal.pone.0315290 (PMC11634006; doi:10.1371/journal.pone.0315290)
Supplement: Attachment — Submitted filename: Comments.docx [file pone.0315290.s006.docx]

In the manuscript titled “The ABA receptor antagonist antabactin restores the germination of thermoinhibited lettuce seeds,” the authors have done an excellent work in understanding the molecular mechanism and solving the problem of seed vigor in lettuce. This work is directly applicable to the agriculture of lettuce and may be further extended to other commercial crops. I have the followings comments and recommendations:

Comments:

1. Figure 1A contains a picture plate, a graph, and the molecular structure of the chemical. Divide these into sub-figures. Nothing is mentioned about the molecule's chemical structure in the text or the figure legend. The authors should mention it in both places.
2. Line 208: Mention both pictures and graphs in-text for readers to easily refer to the data discussed.
3. Line 210: (…32 C?) Remove the question mark and correct the symbol from oC to ^o^C.
4. Line 211, 212, 216: Refer to the figure panel discussed. Make sure that the exact figures are mentioned wherever the data is discussed in-text throughout the manuscript.
5. Line 223: Include a picture plate for the treatment of seeds with AbamineSG similar to the pictures shown for treatment with Fluridone and ANT.
6. Line 243: The correct figure is not cited in this section. Fig S3 and Fig 1D seem interchanged.
7. The color code for the graph in Fig S3 is missing.
8. Line 242-249: Reorganize the data discussed in this section. The data discussed in this section is represented in Fig 1D, Fig S1, and Fig S3. The ordering of figures should match the order of the discussion. Preferably club the two supplementary figures. Also, the values in line 243 and line 244 do not match the graph shown.
9. The authors check the change in expression of LsM3K18A using qRT-PCR. Did they check the expression pattern of 18B and C and was there a reason why only 18A was selected and not 18B and C. I understand that leaves could be used to understand the transcriptional response of ABA but using the seed tissue would give a better picture. The author can use the same RNA as the one used for NCED4 expression analysis to also see the gene expression change of LsM3Ks.
10. Figure 2B is discussed later in the text than 2C. **The order of figures should match the order of the in-text discussion. Check throughout the text.**
11. The author should show the expression pattern of other NCEDs during the thermoinhibition of lettuce seeds.
12. Line 259: Delete (Lsat_1 …..)
13. Line 274: replace out with “our”
14. Divide picture plate and graphs into different subfigure in fig 3. Figure order …..
15. Mention color code for the graph in Figure 3A
16. Line 305: “ the seeds …after-ripened the longest” this might confuse the reader. Author can use something similar to “seeds stored for longer duration since ripening”
