# Supplementary material for: Chemical disruption of ABA signaling overcomes high-temperature inhibition of seed germination and enhances seed priming responses
Source: PLoS One. 2024 Dec 11;19(12):e0315290. doi: 10.1371/journal.pone.0315290 (PMC11634006; doi:10.1371/journal.pone.0315290)
Supplement: Attachment — Submitted filename: Response to Reviewers (3).docx [file pone.0315290.s008.docx]

Response to reviewers

**Reviewer #2:** I am happy with the edits made to the manuscript.

I have few minor recommendations for the manuscript:

1. Line 65-69: Too long statement. Make shorter statements and use proper punctuation for more clarity.

*This has been changed to improve clarity.*

2. Line 81: replace "; it" with "which"

*We have changed this.*

3. Italicize all scientific names

*We have italicized all scientific names.*

4. Methods: Gene expression assay: Mention if extracted RNA was treated for the removal of genomic DNA contamination.

*Yes, we did treat for removal of gDNA and this has now been included in the methods.*

5. Capitalize each word of "Crispr" to "CRISPR" throughout the text.

*We have changed this.*

6. Line 423: change LCMS to LC-MS

*We have changed this and the other mentions of LCMS.*
